# Wear resistance of orthodontic attachments: a comparative analysis of different composite resins in clear aligner therapy

**DOI:** 10.1007/s00784-025-06316-2

**Published:** 2025-04-11

**Authors:** Irmak Ocak, Hande Gorucu-Coskuner, Muge Aksu

**Affiliations:** 1 Department of Orthodontics, Faculty of Dentistry, Hacettepe University, Sihhiye Kampusu, 7. Kat, Altindag, Ankara, Türkiye; 2 Private Practice, Beytepe Mah. , Kanuni Sultan Suleyman Blv, Mira Ofis No: 15/39, Cankaya, Ankara Türkiye

**Keywords:** Attachment wear, Micro-CT, Clear aligner, Conventional attachment

## Abstract

**Objective:**

Clear aligner attachments play a crucial role in facilitating tooth movement in clear aligner treatments. This in-vitro study evaluates the wear of attachments made from 4 different composite resins during orthodontic therapy.

**Materials and methods:**

The study included 32 extracted human premolar and molar teeth, divided into four groups based on the composite resin used: Flow Tain, Transbond XT, G-aenial Universal Flo, and Filtek Z350 XT. Horizontal rectangular attachments were bonded to the teeth, which underwent thermal cycling and tooth brushing to mimic clinical conditions. Micro-CT analysis measured volumetric and linear changes in attachment wear. Statistical analyses were conducted using the Wilcoxon Signed-Rank Test and Kruskal-Wallis test, followed by pairwise comparisons with the Mann-Whitney U Test.

**Results:**

All composite resins exhibited a significant decrease in attachment volume after aging. The greatest volume reduction was observed in the Transbond XT group, while the least reduction occurred in the G-aenial Universal Flo group. Significant differences were found in the occlusal and gingival thirds of the attachments, with Flow Tain showing the highest wear in all regions.

**Conclusion:**

The study shows that the wear of orthodontic attachments is greatly influenced by the type of composite resin used. The results indicate that G-aenial Universal Flo composite resin may offer superior wear resistance, preserving attachment integrity more efficiently throughout orthodontic treatment than the other 3 composite resins.

**Clinical relevance:**

The findings can assist clinicians in choosing composite materials that improve treatment effectiveness by preserving attachment integrity over time.

## Introduction

In contemporary orthodontics, there is an increasing demand for aesthetic treatment options among patients. Clear aligner treatments, initially employed to correct minor irregularities, have now become a preferred choice for addressing various types of malocclusions, owing to technological advancements. These advancements are evident in both the planning software and the materials used during treatment. Consequently, the success rate of clear aligner treatments has improved, leading to a wider adoption of orthodontic procedures that are not only aesthetically pleasing but also comfortable and conducive to maintaining oral hygiene [1, 2].

Attachments, bonded to the tooth surface in different shapes and positions, are used in clear aligner treatments to facilitate tooth movement. The composite resins used in the fabrication of these attachments must satisfy both the aesthetic expectations of patients and the mechanical requirements of the treatment. Given their crucial role in force transmission, it is essential that the attachments maintain their correct position and form. Therefore, careful consideration should be given to both the composition and viscosity of the selected composite resin. Mantovani et al. [3] found a high level of compatibility between various clear aligner applications and different composite resin attachments by using two composite materials with different viscosities, reporting that conventional bulk-fill composite resins produced more successful outcomes. Another study evaluated different composite resins used for attachments and demonstrated that flowable composites resulted in a higher frequency of errors compared to more viscous composites [4]. Conversely, it has also been shown that using low-viscosity composite resins or high-viscosity composite resins in a two-phase procedure leads to more precise results [5].

Surface wear refers to the progressive loss of material over time, which can occur through chemical or physical processes [6]. In this context, the viscosity and particle size of composite resin are critical factors. While an increase in filler content improves mechanical properties, smaller particle sizes enhance aesthetic characteristics [7]. Additionally, the polymerization process and light-curing efficiency significantly impact the surface morphology and wear resistance of composite resin attachments [8]. Surface wear of attachments is a critical consideration, impacting functionality and longevity during orthodontic therapy [9]. A recent study investigated the mechanical properties and wear resistance of different composite resins used for aligner attachments, emphasizing the role of viscosity and filler content in their long-term performance [10]. Barreda et al. [11] observed surface alterations in bulk-fill resins after six months of clear aligner treatment. Li [9] reported that rectangular attachments exhibited the most wear at the gingival edge corners, whereas optimized attachments showed wear across all surface ridges.

Orthodontic treatments are long-term processes, and the wear of attachments over time and the loss of aligner fitting can affect treatment dynamics [12]. Attachments are expected to maintain their full functionality and geometry throughout the course of orthodontic treatment. Otherwise, wear may compromise anchorage control [3]. Therefore, the selection of attachment material is a crucial parameter for the optimization of treatment protocols. The aim of this study is to compare the extent of wear over time in attachments fabricated from different composite resins. The null hypothesis states that there will be no significant difference in surface wear among the composite resins used for attachment fabrication.

## Materials and methods

Our study was ethically approved by the Health Sciences Non-Interventional Research Ethics Committee of Ankara Medipol University (Approval number:60). Extracted human premolar and molar teeth, collected in compliance with the Personal Data Protection Law, were used to form the study groups. The sample size calculation was conducted using G*Power software (version 3.1.9.7; Düsseldorf, Germany). The required sample size per group was determined to be 6 to detect differences in volume loss due to wear between attachments fabricated from two different composite resins (0.59 ± 0.07 mm³; 0.75 ± 0.09 mm³) with an effect size of 1.985, a power of 80%, and an alpha level of 0.05 [13]. To compensate for potential sample loss, 8 samples per group were included, resulting in a total of 32 extracted teeth used in the study. The inclusion criteria for the study were: (1) the absence of any restorations or defects on the tooth surface and (2) proper storage conditions. The exclusion criterion was the presence of material loss on the tooth surfaces.

### Fabrication of clear aligner models

The extracted molar and premolar teeth were first cleaned with a saline solution and subsequently decontaminated using 5.25% sodium hypochlorite. The collection of all teeth took approximately one month, during which they were stored in distilled water at room temperature, with the water being renewed weekly.

The teeth were then embedded in plaster models using a silicone model former to create an arch-like form. The plaster models were scanned and digitized using the Trios 3 scanner (3Shape, Copenhagen, Denmark). Horizontal rectangular attachments, measuring 4 mm in width, 2 mm in height, and 1 mm in thickness, were placed on the premolar and molar teeth in the digital models. Attachment templates (0.4 mm thick, Duran+, Scheu Dental GmbH, Iserlohn, Germany) were obtained from the manufacturer (Orthero software, Şeffaf Aparey Ortodonti ve Elektronik San. Tic. AŞ., Istanbul, Turkiye) and the necessary preparations for bonding were completed.

### Attachment bonding process

The plaster models were randomly assigned to four groups. The groups and the corresponding composite resin protocols are presented in Table 1. Before bonding, all tooth surfaces were cleaned and polished. The buccal surfaces were etched with 37% orthophosphoric acid for 30 s, followed by a 30-second rinse. The etched surfaces were then air-dried for 20 s. Subsequently, the primer application specified in each protocol was performed, followed by the bonding of the attachments using composite resin. Polymerization was carried out using a light-curing device (VALO Ortho Curing Light, Ultradent, South Jordan, UT) at 1000 mw/cm^2^ for 10 s.

**Table 1:** Description of materials used in the study for fabrication of attachments by group

	Primer	Composite Resin Materials	Resin Composition (Matrix)	Resin Composition (Filler)	Resin Filler Content	Manufacturer
Group 1	Assure Universal Bonding Resin	Flow Tain Flowable Composite	Bis-GMA, TEGDMA	Silica, Barium Glass, Fumed Silica	approx. 60% by weight	Reliance Orthodontic Products, Itasca, IL, USA
Group 2	Transbond XT Light Cure Adhesive Primer	Transbond XT Light Cure Adhesive	Bis-GMA, TEGDMA	Silica, Quartz	approx. 70% by weight	3 M Unitek, Monrovia, CA, USA
Group 3	G-Premio BOND	G-aenial Universal Flo	DMA, Bis-MPEPP	Silica, Strontium glass, Fluoroaluminosilicate glass	approx. 69% by weight	GC Corporation, Tokyo, Japan
Group 4	Scotchbond Universal Adhesive	Filtek Z350 XT Flowable Restorative	Bis-GMA, UDMA, TEGDMA, Bis-EMA	Zirconia/Silica	approx. 65% by weight	3 M ESPE, St. Paul, MN, USA

Bis-GMA: Bisphenol A glycidylmethacrylate, TEGDMA: Triethylene glycol dimethacrylate, DMA: Dimethacrylate, Bis-MPEPP: Bisphenol A diglycidyl methacrylate propoxylate, UDMA: Urethane dimethacrylate, Bis-EMA: Ethoxylated bisphenol‑A dimethacrylate, approx: approximately

### In vitro aging protocols

A thermal cycling device (Mod Dental, Ankara, Turkey) was used to simulate the aging process. To replicate a moderate package of clear aligner treatment, 5000 cycles of thermal cycling (submersion in water baths at 5–55 °C for 25 s with a 10-second transition between baths) were performed. Between every 1000 cycles, the models were removed and brushed using the Bass technique with a medium-bristle toothbrush (Colgate 360°, Colgate Sanxiao, China) and a homogeneous mixture of water and toothpaste in a 2:1 ratio (Colgate Total 12, Colgate-Palmolive [China] Company, Guangzhou, China). All brushing procedures were carried out by the same researcher. Assuming that teeth are brushed three times per day for three minutes per session, the brushing time corresponding to one minute for the buccal and occlusal surfaces of posterior teeth was used to calculate the monthly brushing duration for a patient. At the end of each thermal cycling period, 36 min of brushing was performed on the occlusal and buccal surfaces, simulating six months of tooth brushing.

### Micro-CT analysis

After the attachment bonding and aging process, the study models were scanned using a micro-CT device (Skyscan 1275; Bruker Corp., Kontich, Belgium) to perform volumetric and linear measurements. For the attachment analysis, the imaging protocol included a 1-mm aluminum filter, a rotation step of 0.2°, and scanning parameters of 80 kV, 125 µA, with a resolution of 24 μm. In contrast, the protocol for the attachment template analysis did not include a filter but maintained the same rotation step of 0.2°, with scanning parameters adjusted to 40 kV, 250 µA, and a resolution of 26.54 μm. Data reconstruction and artifact avoidance were performed using software (NRecon version 1.6.4.8; Bruker Corp., Kontich, Belgium), generating two-dimensional (2D) cross-sectional images of each specimen. These images were then imported into analysis software (CTan version 1.14.4.1; Bruker Corp., Kontich, Belgium).

**Fig. 1:**
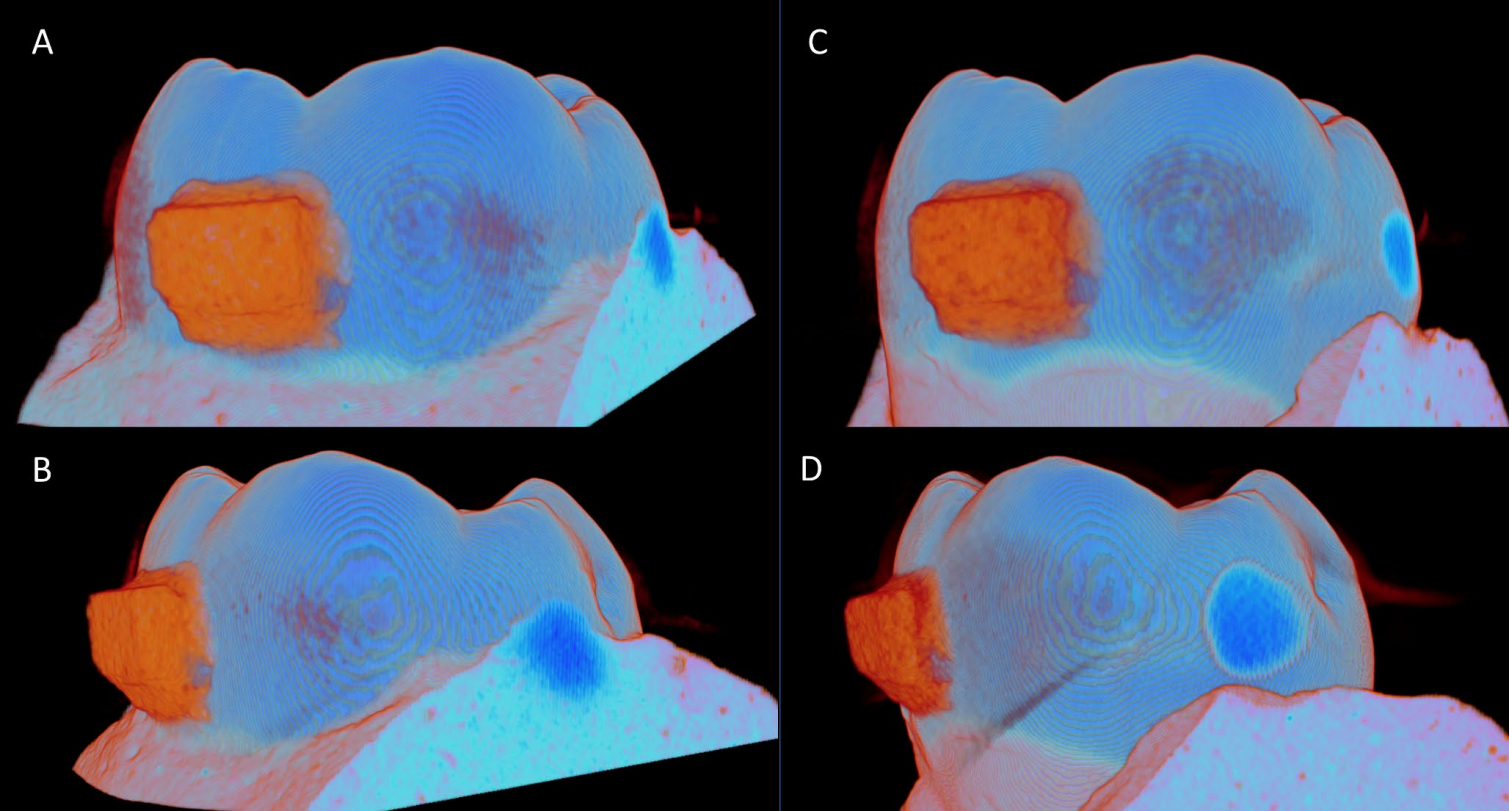
Micro-CT images of the orthodontic attachment before (**A**, **B**) and after (**C**, **D**) aging

The attachment volume was determined by measuring the composite volume remaining on the buccal surface of the tooth before and after aging process. Subsequently, to ensure precise measurement, linear measurements of the composite resin were performed from nine equally spaced points, entering 1 Sect. (24 μm) from the mesial, distal, occlusal, and gingival sides of the attachment. The tooth was divided into three equal regions: occlusal, middle, and gingival. The average of the three linear measurements taken in each section was calculated to determine the lengths of the occlusal, middle, and gingival attachments. Micro-CT images obtained before and after the aging process are presented in Fig. 1.

### Statistical analysis

The normality of the data was assessed using the Shapiro-Wilk test, which indicated that the data did not follow a normal distribution (*p* < 0.05). To evaluate differences between pre- and post- measurements within each group, the Wilcoxon Signed-Rank Test was used. Differences among the four independent groups were analyzed using the Kruskal-Wallis test. When the Kruskal-Wallis test revealed a significant difference between groups, pairwise comparisons were performed using the Mann-Whitney U test. To account for the increased risk of Type I error due to multiple comparisons, the Bonferroni correction was applied to the significance level. The threshold for statistical significance was set at *p* < 0.05.

## Results

The attachment volumes measured before and after the aging process for each group are presented in Table 2. A significant reduction in attachment volume was observed after aging in all composite resins (Table 2). When examining the differences between the groups, the total attachment volume showed statistically significant differences (Table 3, *p* < 0.005). The greatest decrease in attachment volume was observed in the Transbond XT composite resin, while the smallest decrease was observed in the G-aenial composite resin (Table 3). Statistically significant differences were found between the following composite resins: Flow Tain and G-aenial (*p* = 0.021), Transbond XT and G-aenial (*p* = 0.001), and Transbond XT and Filtek Z350XT flowable (*p* = 0.021).

**Table 2:** Comparison of pre- and post-attachment volumes of different composite resins (mm^3^)

	Pre-Attachment Volume	Post-Attachment Volume	*p* value
Group 1 (*n* = 8)	6.53 ± 1.4	5.25 ± 1.15	0.012*
Group 2 (*n* = 8)	7.96 ± 0.97	6.61 ± 1.02	0.012*
Group 3 (*n* = 8)	5.38 ± 1.51	5.03 ± 1.37	0.025*
Group 4 (*n* = 8)	6.69 ± 1.23	6.08 ± 1.14	0.012*

Values are presented as mean ± standard deviation

*p*, Wilcoxon Signed-Rank test; *p < 0.05

**Table 3:** Comparison of attachment volume and attachment lengths differences by different composite resins

	Group 1 (Flow Tain) (*n* = 8)	Group 2 (Transbond XT) (*n* = 8)	Group 3 (G-aenial Universal Flo) (*n* = 8)	Group 4 (Filtek Z350XT flowable) (*n* = 8)	*p* Value^a^	*p* Value^b^
Attachment Volume Difference	1.28 ± 1.14 (19.6%)	1.35 ± 0.64 (16.96%)	0.35 ± 0.35 (6.51%)	0.6 ± 0.38 (8.97%)	0.005**	1–3 *p* = 0,021*, 2–3 *p* = 0,001***, 2–4 *p* = 0,021*
Occlusal Attachment Length	128 ± 29.63	68 ± 40.79	86 ± 22.53	100.38 ± 28.24	0.014**	1–3 *p* = 0,010 **, 1–2 *p* = 0,007**
Middle Attachment Length	97.88 ± 35.6	68 ± 16.56	72 ± 35.26	76 ± 37.03	0.278	
Gingival Attachment Length	128 ± 43.61	88 ± 41.02	56.13 ± 20.9	78 ± 37.71	0.009**	1–3 *p* = 0,001***, 1–4 *p* = 0,021*

Values are presented as mean ± standard deviation. Percentage relative to total attachment volume is shown in parentheses

*p*^a^ Kruskal Wallis test

*p*^b^ Mann Whitney test *p < 0.05, ** *p* < 0.01, *** *p* < 0.001

To evaluate which part of the attachment contributed to the volume difference, linear measurements were assessed. Statistically significant differences were detected between the groups in the occlusal third and gingival third of the attachments (*p* = 0.014 and *p* = 0.009, respectively). In the occlusal third, statistically significant differences were found between the composite resins Flow Tain and G-aenial (*p* = 0.01) and Flow Tain and Transbond XT (*p* = 0.007). In the gingival third, statistically significant differences were observed between the composite resins Flow Tain and G-aenial (*p* = 0.001) and Flow Tain and Filtek Z350XT flowable (*p* = 0.021). The greatest wear was observed in the Flow Tain composite resin in all three regions.

## Discussion

Attachments in clear aligner treatment play a crucial role in both the passive retention of the aligner and the active movement of teeth to achieve treatment objectives [14]. Therefore, to provide adequate support, it is crucial for attachments to maintain their form throughout clear aligner therapy. This study investigated the surface wear of attachments made from different composite resins using micro-CT imaging and found a statistically significant reduction in volume across all composite resins. Additionally, changes in attachment length were observed in the occlusal and gingival regions. The null hypothesis was rejected, as statistically significant differences in surface wear were detected among the different composite resins.

During clear aligner therapy, composite resin is expected to fulfill aesthetic requirements while also possessing sufficient mechanical properties to resist surface wear and fractures. The active surface of the attachment must remain stable to ensure effective force transmission to the tooth in coordination with the aligner as whole [3]. In this study, four different composite resins commonly used in clear aligner therapy were selected, each varying in viscosity and resin filler content. Although flowable composites demonstrate lower durability compared to high-viscosity composites [15], they offer a twofold advantage in terms of working time efficiency [13]. A previous study has demonstrated that the flowability of the composite does not affect attachment shape or volume; however, a significant difference in material overflow was observed [16]. Weckmann et al. [5] reported that low-viscosity composite resins provide higher precision in direct bonding procedures. Consistent with these findings, Olmez et al. [17] reported that fewer bubbles and air voids occur at the bonding surface when using low-viscosity composites. Notably, contemporary low-viscosity composites with high filler content have been observed to exhibit adequate mechanical properties to meet both aesthetic and functional demands throughout the orthodontic treatment duration [5].

In daily routines, various functions such as tooth brushing, eating, and the insertion and removal of clear aligners contribute to attachment wear. Among these, tooth brushing, in particular, involves both the bristles of the toothbrush and the abrasive particles in toothpaste, which mechanically and chemically impact the surface properties of the composite resin [18]. These alterations can lead to increased surface roughness, which not only compromises the aesthetic quality of restorations but also promotes plaque accumulation, potentially resulting in additional dental complications [19]. Previous studies have suggested that increasing the filler content of composite resin improves wear resistance [20, 21]. However, Miyano et al. [22] found no correlation between filler content, viscosity, and wear during tooth brushing. Another study demonstrated that tooth brushing contributes to composite wear, with greater surface degradation observed in composite resins containing larger particle sizes [23]. In that study, polishing was recommended to reduce surface wear following composite resin application. However, it is not feasible to perform polishing after attachment bonding, as from a clinical perspective, it would alter the contact between the attachments and the clear aligner. Therefore, the resin composition and viscosity of the composite resin used for attachment application become crucial factors.

In the present study, a statistically significant reduction in attachment volumes was observed across all composite resins following tooth brushing and aging procedures. Previous studies investigating attachment wear during treatment have similarly demonstrated wear on attachments, consistent with our findings [11, 14, 24]. However, these studies typically utilized a grading system to classify wear severity rather than performing direct measurements. In contrast, Li et al. [9], in their clinical study examining surface wear of different attachment designs throughout treatment, measured attachment volumes and likewise observed that surface wear increased over time. In our study, both volumetric and linear measurements were employed to precisely determine the regions of wear on attachment surfaces, allowing for a detailed comparison across different composite resins.

The greatest reduction in attachment volume, up to 19.6%, was observed in the Flow Tain flowable composite resin, followed by Transbond XT. Wear resistance is influenced by the filler properties, resin matrix formulation, and the bonding between the matrix and fillers. Previous studies have demonstrated that wear resistance can be enhanced by decreasing filler particle size and increasing filler loading [25, 26]. Since Flow Tain has the lowest filler content among the composite resins, this finding is consistent with previous studies. Similarly, Nayyer et al. [21] found that composite resins with low filler content exhibited lower microhardness and greater wear due to tooth brushing. Interestingly, despite its medium flowability and high filler content, Transbond XT exhibited a high degree of wear. This is thought to be due to the composition of the composite resin.

The composite resin G-aenial, which exhibited the least wear, differs compositionally from the other composite resins. Unlike the others, G-aenial does not contain TEGDMA. While Bis-GMA provides viscosity to the composite resin, TEGDMA is used as a diluent, which can result in undesirable effects such as increased water absorption, reduced physical properties, and decreased color stability [22]. Additionally, although Bis-MPEPP, which is present in G-aenial, has lower viscosity than Bis-GMA, it offers greater long-term durability due to its lower water absorption and enhanced surface homogeneity. This likely explains its superior long-term performance after tooth brushing and aging. Another composite resin with low wear is Filtek Z350XT, which, despite being a flowable composite, contains a high filler content. The UDMA present in Filtek Z350XT has been shown to provide better wear resistance compared to Bis-GMA-based composite resins [9, 27].

Linear measurements were conducted to precisely determine which regions were affected by volumetric changes. The reduction in attachment dimensions was most pronounced with the Flow Tain composite resin across all tooth surfaces, while the least reduction occurred with the G-aenial composite resin. Barreda et al. [11] demonstrated that the use of different composites could influence the surface properties of attachments but not their overall shape. However, in their study, surface changes were assessed through simple visual classification without quantitative measurement. Our findings revealed that wear in composite attachments was statistically significantly greater in the occlusal and gingival thirds, indicating that changes primarily occur in the angular regions of the attachment. Fausto et al. [24] reported that surface wear in attachments, particularly in conventional attachments on anterior teeth and on the distal side, was more pronounced after 4–6 months. Contrary to our findings, they observed greater wear in the central regions of the attachments, with a reduction towards the edges, and concluded that these changes were not clinically significant. However, their study utilized only a single brand of high-viscosity composite resin for all attachment productions. In another study, Filtek Z350 XT composite resin was used, and consistent with our findings, the greatest wear in a 3 mm rectangular attachment design was observed at the gingival corner edges. This evaluation was conducted using a three-dimensional superimposition method [9]. In their study, Vas and Varghese [14] investigated the effects of toothbrushes with varying bristle hardness on the surface of attachments made with Transbond XT composite resin. They reported that the most significant morphometric changes occurred with hard and medium-hard toothbrushes. However, the attachment bonding template used in their study was not the same as those typically used in clinical practice. As a result, it is possible that the surface characteristics of the attachments prepared using a system where the composite surface was exposed may vary.

In our study, which investigated the surface changes in attachments produced with different composite resins after tooth brushing, it was observed that the surface structure of the attachments did not remain stable and was subjected to wear. These morphological changes in the attachments could potentially reduce the aligner’s gripping ability, thereby compromising the effectiveness of achieving the desired tooth movements. However, it is important to acknowledge the limitations of our study. One of the primary limitations is the in vitro nature of the study, which may not fully replicate the intraoral environment. Additionally, the relatively small sample size may restrict the generalizability of our findings, and a larger sample could yield more robust conclusions. Furthermore, the results may also vary depending on composite resins with different compositions and viscosities, as well as on attachments with alternative designs not included in this study. Therefore, it is crucial to recognize that the choice of composite resin is a significant parameter in clear aligner treatment. Future studies should focus on comprehensive evaluations of the parameters influencing attachment production and their impact on tooth movement outcomes.

## Conclusion

In this study, the G-aenial composite resin demonstrated the highest wear resistance among the tested materials. The most significant wear was observed in the occlusal and gingival regions of the attachments. This wear could potentially reduce the aligner’s effectiveness in retention and tooth movement. Therefore, the selection of composite resin is crucial for maintaining attachment integrity and ensuring the success of clear aligner therapy.

## Data Availability

No datasets were generated or analysed during the current study.
